# Apathy and Emotion-Based Decision-Making in Amnesic Mild Cognitive Impairment and Alzheimer's Disease

**DOI:** 10.1155/2014/231469

**Published:** 2014-06-22

**Authors:** Sophie Bayard, Jean-Pierre Jacus, Stéphane Raffard, Marie-Christine Gely-Nargeot

**Affiliations:** ^1^Centre d'Investigation Clinique, Centre Hospitalier Universitaire Montpellier, 80 avenue Augustin Fliche, 34295 Montpellier Cedex 5, France; ^2^Laboratoire Epsylon, EA 4556, Université Montpellier, rue du Professeur Henri Serre, 34000 Montpellier, France; ^3^Consultations Mémoire, CH Val d'Ariège, BP 01, 09017 Foix, France; ^4^Service Universitaire de Psychiatrie Adulte, Centre Hospitalier Universitaire Montpellier, 39 avenue Charles Flahault, 34295 Montpellier Cedex 5, France

## Abstract

*Background*. Apathy and reduced emotion-based decision-making are two behavioral modifications independently described in Alzheimer's disease (AD) and amnestic mild cognitive impairment (aMCI). *Objectives*. The aims of this study were to investigate decision-making based on emotional feedback processing in AD and aMCI and to study the impact of reduced decision-making performances on apathy. *Methods*. We recruited 20 patients with AD, 20 participants with aMCI, and 20 healthy controls. All participants completed the Lille apathy rating scale (LARS) and the Iowa gambling task (IGT). *Results*. Both aMCI and AD participants had reduced performances on the IGT and were more apathetic compared to controls without any difference between aMCI and AD groups. For the entire sample, LARS initiation dimension was related to IGT disadvantageous decision-making profile. *Conclusions*. We provide the first study showing that both aMCI and AD individuals make less profitable decisions than controls, whereas aMCI and AD did not differ. Disadvantageous decision-making profile on the IGT was associated with higher level of apathy on the action initiation dimension. The role of an abnormal IGT performance as a risk factor for the development of apathy needs to be investigated in other clinical populations and in normal aging.

## 1. Introduction

Reduced decision-making performances on the IGT have been described in various neurodegenerative diseases such as Parkinson's disease, Alzheimer's dementia, and frontotemporal and Huntington diseases [1]. More recently, poor decision-making performances were also described in patients who are at a greater risk of developing dementia compared with healthy older adults, that is, MCI [2] and idiopathic rapid eye movement sleep behaviour disorder [3, 4]. Apathetic manifestations are very common across these neurological conditions and have been associated with a wide range of negative consequences for the patients and the caregivers [5–7]. Despite the important prevalence of this problematic manifestation, the underlying mechanisms of apathy are still poorly understood hindering the development of targeted and effective rehabilitation. With this background, the emotion-based decision-making as assessed by the IGT is an attractive concept to understand neurocognitive correlates of apathy. To the best of our knowledge, only one study has documented that the IGT is sensitive to the presence of apathy symptoms in brain damaged patients [8].

In the present study, we aimed to assess whether disadvantageous decision-making profile on the IGT is associated with apathy symptoms in patients affected by AD and in participants with amnesic MCI (aMCI) compared to healthy controls. We hypothesized that both AD and MCI participants would perform more disadvantageously on the IGT compared to healthy controls. Furthermore, we hypothesized that a higher level of apathy would be associated with poorer decision-making performances.

## 2. Material and Methods

### 2.1. Participants

Participants with AD (*n* = 20) and aMCI (*n* = 20) were recruited from a memory clinic located in the Centre Hospitalier du val d'Ariège (France) where they were diagnosed by an experienced clinical neurologist after neuropsychological testing. In addition, AD and aMCI participants went through an extensive medical, neurological, and neuroradiological examination to exclude the presence of any other significant systemic, neurological, or psychiatric disorder that could explain their cognitive difficulties.

A diagnosis of aMCI was made by a senior neurologist and a clinical neuropsychologist Jean-Pierre Jacus using specific operational criteria [9] which included (1) memory complaint corroborated by an informant, (2) impaired episodic memory, (3) normal general cognitive function as determined by a clinician's judgment and based on a structured interview with the patient and an informant, (4) intact activities of daily living as determined by a clinician's judgment and a structured interview with the patient and an informant, (5) and not fulfilling National Institute of Neurological and Communicative Disorders and Stroke/AD and Related Disorders Association criteria for AD [10]. None of the participants had a pathological gambling problem. Twenty patients were diagnosed as having probable AD according to NINCDS-ADRDA criteria [10].

Controls participants (*n* = 20) were healthy community-dwelling adults living in Ariège (France). All were native French speakers with normal or corrected hearing and vision at the time of testing. They were recruited from an adult participants' pool and senior-citizen associations. Each participant was screened for neurological and psychiatric disorders that could affect cognitive abilities. Interviews were conducted by a registered psychologist Jean-Pierre Jacus. None of the controls had a history of neurological or psychiatric illness and had a Minimental State Examination score above the 10th percentile taking into account their level of education [11].

All participants underwent psychiatric, neurological, and physical examinations. Participants with a history of alcoholism, head injury, epilepsy, Parkinson's disease, major medical disease, major psychiatric disorder, or significant vascular risk factors were excluded. None of the participants were a priori excluded for mood disorders. All completed the 21-item Beck depression inventory. Prior to the study, all procedures were explained to the participants and their written consent was obtained. All protocols were approved by Paul Valery University Institutional Review Board. Participants did not receive any incentive or honoraria in exchange for their participation.

### 2.2. Lille Apathy Rating Scale

The LARS is self-report questionnaire (structured interview) on thoughts, emotions, and activities over the previous four weeks [12]. It contains 33 items. The LARS comprises four subscales corresponding to the four distinct yet related facets of apathy identified by Sockeel and colleagues: intellectual curiosity (lack of interest, novelty seeking and motivation, and poor social life), emotion (blunting of emotional responses and lack of concern), action initiation (low everyday productivity and lack of initiative), and self-awareness. The total score can range from −36 (optimal) to +36 (worst score). We used also a cutoff value according to the four levels of apathy [12]. Participants were classified into two categories: “nonapathetic” (−36 to −22) versus “slightly apathetic to severely apathetic” (−21 to 36).

### 2.3. Iowa Gambling Task

The IGT was administered to assess decision-making under ambiguity [13]. In this task, participants are instructed that the goal of the game is to win as much fictitious money as possible. The task entailed a series of 100 card selections from four decks (A, B, C, and D), but participants are not informed of the number of trials. Although they are told that some card decks might be better than others, they do not know which ones are advantageous or disadvantageous. Decks A and B are classified as disadvantageous because the final balance is negative, with high immediate gains of money but even higher future losses. In contrast, selecting a card from decks C and D produces small gains, but unpredictable losses are smaller, so that these decks will reward more money in the long run and are thus considered to be advantageous. Quantitative outcomes consist in the net win € and the net score (selection from decks C and D minus selections from decks A and B) computed both for the whole 100 cards (total net score) and for the five successive blocks of 20 cards each (1–20, 21–40, and so on). This later outcome is used in order to quantify the progressive change in selection across the IGT. A positive net score indicates more frequent selection from advantageous decks (i.e., advantageous profile), whereas a negative net score (IGT < 0) indicates more frequent selection from disadvantageous decks (i.e., disadvantageous profile). We also made a distinction between the initial phase (trials 1–40; blocks 1 and 2) and the second part of the IGT (trials 41–100; blocks 3, 4, and 5) [14].

### 2.4. Executive Function Assessment

#### 2.4.1. Trail Making Test

This test assesses visual scanning ability, processing speed, and set-shifting/executive functioning and is coded as the number of seconds needed to correctly complete connection of the number and number-letter sets [15]. In the last condition of the test (condition 4), Number-Letter Switching requires the participant to connect alternately numbers and letters in order (e.g., 1-A-2-B-3-C) and serves as the primary measure of executive function for this test. During the test, errors made during the sequencing conditions were pointed out to the participants and they were asked to return to the last correct connection and continue from there. The two dependent variables were Number-Letter Switching completion time and error score from condition 4.

#### 2.4.2. Hayling Test

The Hayling Test evaluates initiation speed as well as automatic response inhibition [16]. It requires the subject to complete 15 sentences by filling in the correct missing word (automatic condition) and a nonsense word (inhibition condition) and provides an index of response initiation and suppression. Two composite scores were computed (inhibition condition and automatic condition) for completion time and error score.

#### 2.4.3. Updating Memory Task

This task taps the ability to monitor and code incoming information for relevance to the task at hand and then appropriately revise the items held in working memory by replacing old, no longer relevant information with newer, more relevant information [17]. The dependent variable was calculated by averaging the number of consonants correctly (serially) remembered over all span levels.

### 2.5. Statistical Analyses

The data were examined for normal distribution (tested with Kolmogorov-Smirnov test) and homogeneity of variance (tested with the Levene test). For normal distributed data, parametric tests were used (Student's* t*-test for independent samples, univariate analysis of covariance (ANCOVA), analysis of covariance with repeated measures, and Greenhouse-Geisser adjusted degrees of freedom (MANCOVA)).

In case of significant deviations from the normal distribution, we used corresponding nonparametric methods (Mann-Whitney* U* test, chi-square test, and logistic regression). We calculated partial eta squared (*η* ^2^) and Cohen's *d*′ as a measure of the effect size and designated the effect size as small (*η* ^2^ = 0.01; *d*′ = 0.2), medium (*η* ^2^ = 0.06; *d*′ = 0.5), or large (*η* ^2^ = 0.14; *d*′ = 0.8). The level of significance was set at *P* < 0.05. All statistical analyses were carried out using the Statistical Package for the Social Sciences (SPSS) version 19 for Windows.

## 3. Results

### 3.1. Demographic and Clinical Data

Demographic and clinical data are reported in Table 1. AD and aMCI participants were older (resp., *P* < 0.001 and *P* = 0.043) and less educated (resp., *P* = 0.09 and *P* = 0.027) than controls, without any difference between AD and aMCI participants (all *P* values = 1). Groups were matched for gender. As MMSE total score was associated with both age (rho = −0.42, *P* = 0.001) and education (rho = 0.41, *P* = 0.001), MMSE of the three groups were compared with these two demographic variables as covariates. As expected, AD participants performed worse than aMCI participants (*P* = 0.006) and controls (*P* < 0.001) on the MMSE, as significant difference was also observed between aMCI participants and controls (*P* = 0.018). Finally, there was no significant difference between groups in self-reported depression symptom severity using the BDI total score or different clinical cutoff scores.

### 3.2. Lille Apathy Rating Scale

As the LARS was originally validated in patients with Parkinson's disease, we decided to compute its internal consistency in terms of Cronbach's standardized *α* coefficient. The LARS was found to be reliable (33 items; *α* = 0.77). The Cronbach's *α* value is acceptable.

When controlling for age and education, between-group differences were observed for the LARS dimensions intellectual curiosity (*P* = 0.006, *η* ^2^ = 0.17), action initiation (*P* = 0.009, *η* ^2^ = 0.15), and the total score (*P* < 0.001,  *η* ^2^ = 0.25) (Table 1). For all of these dimensions, contrast analyses revealed that aMCI and AD participants scored significantly higher (i.e., more apathetic) than controls (all *P* values <0.05, all Cohen *d*′ > 0.8). No significant difference was noted for emotion (*P* = 0.15) and self-awareness (*P* = 0.86) dimensions. Using the LARS cutoff scores, 5% of controls, 60% of aMCI, and 60% of AD participants were classified as slightly to severely apathetic (*P* < 0.001, OR = 3.62, 95% CI 1.6 to 7.7). This association persisted after adjustment for age and education (*P* = 0.006, OR 1.1, 95% CI 1.02 to 1.30).

### 3.3. Iowa Gambling Task

**Figure 1:**
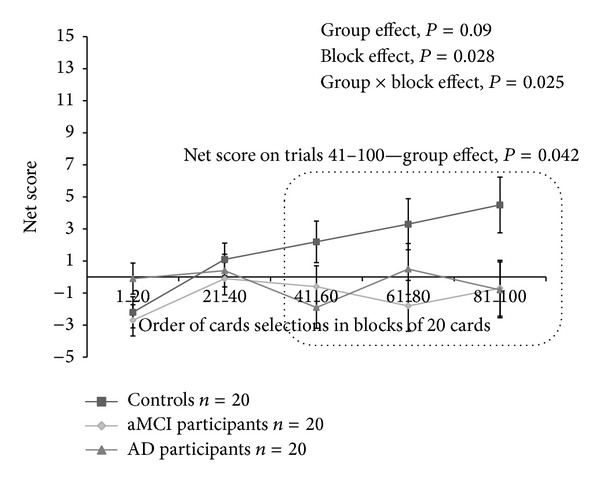
Iowa gambling task net scores for the 5 blocks, consisting of 20 cards selections for controls, aMCI, and AD participants. Mean (±SEM) are given.

In groups and in the whole sample, we found no association between age, education, and IGT variables. A group effect was observed for the IGT net win (*F* = 6.83, *P* = 0.002) with lower final outcome in aMCI (mean = 492, SD = 255) and AD participants (mean = 630, SD = 199) compared to controls (mean = 1416, SD = 280; resp., *P* = 0.003, Cohen *d*′ = 3.63 and *P* = 0.014, Cohen *d*′ = 3.27). Additionally, we found no difference between aMCI and AD patients (*P* = 0.1). To analyze the IGT performance in more detail, we conducted a 3(group) × 5(block) repeated-measures ANOVA on the IGT net score. There was significant block (*F* = 3.025, *P* = 0.028, *η* ^2^ = 0.05) and an interaction group × blocks between controls, aMCI, and AD patients (*F* = 2.42, *P* = 0.025, *η* ^2^ = 0.07), indicating that aMCI and AD participants and controls displayed different decision-making patterns during the task (Figure 1). With respect to the net score for trials 41 to 100, a group effect was noted (*F* = 3.43, *P* = 0.042, *η* ^2^ = 0.10). Controls performed better than aMCI and AD patients (resp., *P* = 0.048, Cohen *d*′ = 1.18 and *P* = 0.043, Cohen *d*′ = 1.21). No difference was observed between aMCI and AD patients (*P* = 0.8). Finally, according to their performances at the IGT total net score and block 3 to 5 net score (trials 41–100), the proportion of participants with IGT disadvantageous profile (net score < 0) was higher in aMCI and AD groups compared to controls (Table 1, all *P* values <0.05). No significant difference was observed between AD and aMCI participants (all *P* values >0.8).

### 3.4. Executive Function Assessment

As reported in Table 1, the ANCOVA with age and education as covariates indicated difference between groups for error score on the Hayling Test (*F* = 3.27, *P* = 0.04, *η* ^2^ = 0.10) and for time completion on the Trail Making Test (*F* = 5.98, *P* = 0.004, *η* ^2^ = 0.17). Both AD and MCI participants were slower than controls on the Trail Making Test (resp., *P* = 0.003 and *P* = 0.005). AD and MCI participants' error rate in the Hayling Test was higher than that of control participants (resp., *P* = 0.02 and *P* = 0.03). We found no significant difference between AD and MCI groups for these two executive indices (all *P* values >0.70).

### 3.5. Iowa Gambling Task and Lille Apathy Rating Scale

**Figure 2:**
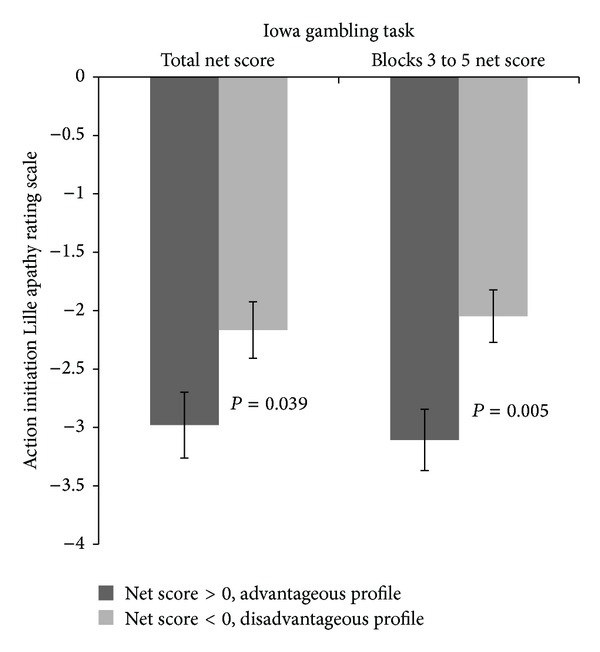
Lille Rating Scale's action initiation score for participants (*n* = 60) with advantageous and disadvantageous profile on the total net score and on the net score for blocks 3 to 5 (trials 41–100). Mean (±SEM) are given.

A two-factor within subjects ANOVA 3(group) × 2(IGT profile) performed on LARS dimensions and total score indicated a significant group effect for all LARS indices (all *P* values <0.05) without any group × IGT interaction. Given this lack of interaction, the relationship between decision-making performances and apathy was studied with one-way ANOVA without stratifying participants as according to their group. According to their performances at the IGT total net score, 29 participants were identified to have a disadvantageous profile (IGT < 0); 28 demonstrated a preference for disadvantageous choices for blocks 3 to 5 net score (trials 41–100; IGT < 0).  Figure 2 indicated that participants with advantageous profile at the IGT (net score > 0) were less apathetic than participants who demonstrated a preference for disadvantageous choices (net score < 0) only on the LARS action initiation dimension (total net score, *P* = 0.039, Cohen *d*′ = 0.59; net score for blocks 3 to 5, *P* = 0.005, Cohen *d*′ = 0.79). Between-group comparisons for intellectual curiosity (resp., *P* = 0.29), emotion (resp., *P* = 0.18), self-awareness (resp., *P* = 0.82) dimensions, and the total score (resp., *P* = 0.07) did not reach significance.

Decision-making profiles on IGT (i.e., advantageous versus disadvantageous) were not associated with any demographical variables, executive performances, or self-reported depressive symptoms (all *P* values >0.50).

## 4. Discussion

The main results of this study (i) confirm altered decision-making in aMCI and AD as examined by the IGT and (ii) indicate, for the first time, in a single study, that aMCI and AD participants have similar disadvantageous decision-making profile on the IGT and (iii) similar levels of apathy; (iv) finally, we show that a higher level of apathy on the LARS action initiation dimension appears to be related to IGT disadvantageous decision-making profile.

In comparison to healthy controls, aMCI and AD participants made more disadvantageous choices. Both groups opted more frequently than healthy controls for decks with high immediate reward regardless of higher future punishment failing to increase adaptability. We also found that participants with aMCI performed at the same level as participants with AD on the IGT. Up to now, very few studies have studied decision-making processing in aMCI and AD, while these populations have to take important decisions despite their cognitive disorders. Patients with AD typically have disadvantageous profiles on the IGT [18]. Reduced decision-making performances on gambling tasks with stable and explicit rules, that is, the game of dice task and the probability-associated-gambling task, were also documented in this neurodegenerative disease [19, 20]. Performance on the IGT has been attributed to dysregulations of somatic markers [21]. Namely, individuals who perform poorly on this task purportedly have weaker somatic or physiological (i.e., emotions) cues to guide risky choices, leading to a “myopia for the future” [22]. Brain lesion studies have confirmed that the limbic loop is involved in reward processing as measured by the IGT [23]. This paradigm has been also recognized as sensitive to psychiatric conditions such as substance dependence and abuse, pathological gambling, and PD with mesolimbic and mesocortical circuit alterations [24]. In the early stages of AD, degeneration occurs in the medial temporal lobes. As the disease progresses, other brain areas, such as the lateral temporal, frontal, and parietal cortices, are typically affected [25]. In AD patients, Chu and coworkers documented severe pathological changes in the ventromedial frontal mesocortical regions providing evidence about possible neurobiological substrates for change in emotion behaviour and autonomic function in this pathology [26]. These changes in the ventromedial frontal cortex were associated with autonomic dysfunctions. Although aMCI is a controversial clinical entity, it remains associated with an increased risk of dementia [27]. Even though the neuropathologic substrates of aMCI are complex and still unanswered, postmortem studies frequently reported structural and neurochemical alterations in cortical and basal limbic forebrain regions [28]. In this context, we suggest a direct involvement of AD and aMCI neuropathologic modifications in IGT disadvantageous decision-making profile observed in these two clinical conditions. Our findings are consistent with the early pathological cerebral changes and related (cognition and emotional) alterations reported in both aMCI and AD conditions.

Even if apathy has been frequently described as a risk factor of faster conversion from aMCI to AD [29], no study has directly assessed apathy severity in AD and aMCI in comparison with healthy controls. By using a multidimensional approach of apathy with the LARS, we show that aMCI and AD participants reported a higher level of apathy symptoms than controls without any significant difference between AD and aMCI. Interestingly, this higher level of apathy was observed in the context of nonsignificant group's difference for self-reported depression symptom severity. Our results are in accordance with the notion that apathy is a common symptom in aMCI and AD [6, 29]. Furthermore, our results confirm that apathy may occur in the absence of depression in participants with aMCI and AD [30]. In the present study, none of participants were a priori excluded for mood disorder and particularly for major depression. Finally, from a methodological perspective, we confirmed the clinical value of the LARS in apathy evaluation. In fact, we found that both aMCI and AD conditions increase the risk of scoring above the LARS clinical cutoff (i.e., “nonapathetic” versus “slightly apathetic to severely apathetic”) compared to healthy controls.

To our knowledge, this study is the first to identify a relationship between decision-making performance and apathy. As we found no significant group × IGT profile interaction on the LARS, we decided to study the relationship between IGT profile and LARS dimensions on the whole sample. Participants with a disadvantageous decision-making profile were more apathetic on the LARS action initiation dimension than participants who demonstrated an advantageous profile. Interestingly, we found no association between IGT and executive performances. Several studies have proposed definitions which incorporate distinct components of apathy (behavioral, cognitive, and emotional) [31]. Behavioral apathy related to an autoactivation deficit refers to difficulties in activating thoughts or initiating the motor program necessary to complete the behavior. As suggested by Levy and Dubois [31] conceptualization, this form of apathy results from “a failure to reach the threshold of initiation/activation of thoughts or actions when subjects should behave on an internal basis but not in externally driven responses.” From a pathophysiological point of view, apathy results from a dysfunction of the limbic circuit connecting the ventral striatum to orbitofrontal and anterior cingulate cortex. The impact of this dysfunction leads to a loss of temporal and spatial focalization, both of which result in a diminished extraction of the relevant signal with the frontal cortex, thereby inhibiting the capacity of the frontal cortex to select, initiate, maintain, and shift programs of action. Three main systems are thought to be involved in decision-making: a stimulus encoding system supported by the orbitofrontal cortex, an action selection system relying on the anterior cingulate cortex, and an expected reward system depending on basal ganglia and amygdala [1]. According to their definition, neither LARS intellectual curiosity, emotion, nor self-awareness dimensions tap the three main systems involved in decision-making. However, the LARS action initiation refers to the notions of low everyday productivity and lack of initiative that could reflect a disruption in the action selection system when behavior needs to be internally driven (i.e., behavioral apathy). This disruption could be reinforced by the ambiguous nature of external cues provided by the IGT when losses/gains feedback is given to participants.

Several limitations in our study need to be addressed. One is the difference in education and in age between groups. However, we found no association between age, education, and IGT performances. Another limitation is that our results are based on a cross-sectional approach, which do not indicate causality. In fact, the link between apathy and decision-making as assessed by the IGT is presumably bidirectional: some aspects of apathy may contribute to the inability to accurately evaluate the consequences of choices and actions, thus inducing a quantitative decrease in decision-making. In the context of the present study, the question remains as to whether propensity towards disadvantageous choices on the IGT constitutes a potential risk factor to further develop apathetic symptoms in both aMCI and AD populations. Longitudinal studies of elderly subjects may demonstrate whether reduced IGT performance may predispose to the development of apathy. Finally, we are aware that our sample size is small. This statistical limitation might explain the similar IGT decision-making profiles of AD and aMCI.

In conclusion, we provide here the first study describing that both aMCI and AD individuals make less profitable decisions relative to controls, without difference between aMCI and AD. We also documented a relationship between disadvantageous decision-making profile on the IGT and higher level of apathy on action initiation dimension. Finally, the role of an abnormal IGT performance as a risk factor for the development of apathy needs to be investigated in other clinical populations (e.g., PD and schizophrenia) and in normal aging.

## Figures and Tables

**Table 1:** Demographic and clinical characteristics and Lille Apathy Rating Scale rates for AD and MCI participants and controls.

	Healthy controls (n = 20)	MCI participants (n = 20)	DTA participants (n = 20)	Statistics	*P* value
Demographic and clinical data					
Age, mean (SD)	73.5 (6.7)	78.25 (6.9)	80.9 (5.4)	*F* = 8.7	<0.001^a^
Sex, *n* (women)	11	11	12	*χ* ^2^ = 0.13	0.93
Years of education, mean (SD)	11.1 (2.7)	7.9 (2.4)	8.3 (3.1)	*F* = 7.7	0.001^a^
Minimental State Examination^c^, mean (SD)	28.5 (0.9)	27.15 (2)	24.8 (2.3)	*F* = 11.6	<0.001^b^
Beck depression inventory					
Total score, mean (SD)	10.6 (6.5)	12.2 (7.8)	13 (7.44)	*F* = 0.57	0.57
Moderate (>18), *n* (%)	2 (10)	4 (20)	5 (25)	*χ* ^2^ = 1.55	0.45
Severe (>19), *n* (%)	1 (5)	0	0	*χ* ^2^ = 2.03	0.36
Lille apathy rating scale^c^, mean (SD)					
Intellectual curiosity	−2.6 (0.76)	−1.4 (0.96)	−1.5 (1.01)	*F* = 5.6	0.006^a^
Emotion	−3.6 (0.59)	−2.5 (1.29)	−2.7 (1.50)	*F* = 1.9	0.15
Action initiation	−3.5 (0.64)	−2.2 (1.15)	−2.0 (1.74)	*F* = 5.1	0.009^a^
Self-awareness	−2.9 (1.14)	−2.4 (1.27)	−2.5 (1.31)	*F* = 0.1	0.86
Total score, mean (SD)	−28 (4.18)	−17.8 (6.80)	−19.1 (6.40)	*F* = 9.6	<0.001^a^
					
Lille apathy rating scale-cutoff					
Lightly apathetic to severely apathetic, *n* (%)	1 (5)	12 (60)	12 (60)	OR = 3.62 95% CI 1.6 to 7.7	<0.001
IGT disadvantageous profile (net score <0)					
Total net score, *n* (%)	6 (20)	13 (45)	10 (35)	*χ* ^2^ = 5.64	0.05^a^
Blocks 3 to 5 (trials 41–100), *n* (%)	5 (18)	12 (42)	11 (39)	*χ* ^2^ = 6.66	0.03^a^
					
Executive function assessment^c^, mean (SD)					
Hayling Test (time)	4 (2.34)	7.5 (4.51)	6.8 (3.67)	*F* = 1.66	0.19
Hayling Test (error)	2.7 (1.92)	8.5 (7.34)	9.8 (5.90)	*F* = 3.27	0.04^a^
Trail Making Test (time)	112 (50)	191 (60)	198 (46)	*F* = 5.98	0.004^a^
Trail Making Test (error)	0.5 (1.05)	3.1 (4.58)	5.3 (7.14)	*F* = 1.96	0.14
Updating memory task	3.3 (0.83)	2.6 (0.64)	2.4 (0.64)	*F* = 1.56	0.21

AD: Alzheimer's disease; CI: confidence internal; IGT: Iowa gambling task; MCI: mild cognitive impairment.

^a^Controls *≠* (MCI = DTA); ^b^(Controls = MCI) *≠* DTA; ^c^adjustment for age and education.
